# The overlap between autistic spectrum conditions and borderline personality disorder

**DOI:** 10.1371/journal.pone.0184447

**Published:** 2017-09-08

**Authors:** Robert B. Dudas, Chris Lovejoy, Sarah Cassidy, Carrie Allison, Paula Smith, Simon Baron-Cohen

**Affiliations:** 1 Autism Research Centre, Department of Psychiatry, University of Cambridge, Cambridge, United Kingdom; 2 Cambridge City Older People’s Mental Health Service, Cambridgeshire and Peterborough NHS Foundation Trust, Cambridge, United Kingdom; 3 Centre for Research in Psychology, Behaviour and Achievement, Coventry University, Coventry, United Kingdom; 4 CLASS Clinic, Cambridgeshire and Peterborough NHS Foundation Trust, Cambridge, United Kingdom; Universite de Bretagne Occidentale, FRANCE

## Abstract

**Background:**

Both people with autism spectrum conditions (ASC) and borderline personality disorder (BPD) are significantly challenged in terms of understanding and responding to emotions and in interpersonal functioning.

**Aims:**

To compare ASC, BPD, and comorbid patients in terms of autistic traits, empathy, and systemizing.

**Methods:**

624 ASC, 23 BPD, and 16 comorbid (ASC+BPD) patients, and 2,081 neurotypical controls (NC) filled in the Autism Spectrum Quotient (AQ), the Empathy Quotient (EQ) and the Systemizing Quotient-Revised (SQ-R).

**Results:**

On the AQ, the ASC group scored higher than the BPD group, who in turn scored higher than the comorbid group, who scored higher than controls. On the EQ, we found the comorbid and ASC groups scored lower than the BPD group, who were not different from controls. Finally, on the SQ-R, we found the ASC and BPD group both scored higher than controls.

**Conclusions:**

Similar to ASC, BPD patients have elevated autistic traits and a strong drive to systemize, suggesting an overlap between BPD and ASC.

## Introduction

Autism Spectrum Conditions (ASC) are diagnosed by the presence of social and communication difficulties, alongside unusually strong, narrow interests and/or unusually repetitive and stereotyped behaviour (DSM-5, [1]. We prefer the term ASC rather than ASD (Autism Spectrum Disorder) because it is less stigmatising. Also, ASC is more consistent with the fact that these individuals have not only disabilities requiring a medical diagnosis, but also areas of cognitive strength. Autistic traits are continuously distributed in the general population, and the threshold for diagnosis is determined by clinical judgement. The prevalence of ASC is estimated at 1% and is more often diagnosed in males [2].

Borderline Personality Disorder (BPD) has a lifetime prevalence of 5.9% and is more often diagnosed in females [3] and, according to DSM-5 [1], is characterised by impairment in interpersonal functioning (including poor empathy, and problems with trust and intimacy) [4] and difficult personality traits, such as disinhibition and antagonism, and impulsivity [5–8].

The exact aetiology, including the contribution of genetic and environmental factors, of ASC or BPD is not known, and even less is known about the psychopathological relationship between ASC and BPD. The symptomatic overlap of ASC and BPD has been noted for some time [9,10] and at the cognitive level, ASC includes difficulties in reading others’ emotions [11,12] and core cognitive features of BPD also include altered social cognition [13,14].

To our knowledge, only one study has compared BPD and ASD on personality traits [15], which used the NEO-Personality Inventory-Revised (NEO-PI-R) and found more neuroticism, extraversion, and openness for experience but less conscientiousness and the same level of agreeableness in BPD vs. ASC. They also found, using the Dimensional Assessment of Personality Pathology Questionnaire (DAPPBQ), more emotional dysregulation and dissocial behaviour and less inhibition and compulsivity in BPD vs. ASC. Looking at the items of these subscales, there was no difference between the two groups in terms of intimacy, social avoidance, restricted expressiveness and callousness. The symptomatic overlap of ASC and personality disorders can lead to differential diagnostic uncertainty, particularly in women [16–19]. Looking at patients with Asperger Syndrome, Hofvander et al. found that 42 out of 62 (68%) met DSM-IV criteria for at least one personality disorder and, similarly, Lugnegard et al. found 26 out of 54 (48%) did. Conversely, Ryden [20] looked for ASC in BPD patients and found that 6 out of 41 BPD patients fulfilled criteria for ASC.

Correct clinical diagnosis is important, as the existing relatively small amount of evidence, especially as regards adult autism, suggests that different intervention approaches might be effective in the two conditions. For example, self-harm in ASC has been found to be strongly associated with sensory overload [21], while in BPD it tends to occur in the context of interpersonal conflict and emotional dysregulation. Thus, in ASC it may seem reasonable to manage it by reducing activities causing sensory overload, while in BPD there is evidence for the efficacy of psychological interventions that reduce emotional dysregulation or prompt ‘mentalizing’ [22,23]. In addition, patients can be diagnosed with both ASC and BPD, and there is some indication that this comorbid population may be at increased risk of suicide [20], which further highlights the importance of appropriate diagnosis.

In the present study we aimed to investigate 3 areas of psychological functioning in order to identify symptomatic signatures in these patient groups: autistic traits, empathizing, and systemizing. The first two of these are well established. The construct of systemizing is defined as the drive to analyse or build a system, which itself is defined as any rule-based pattern of information [24]. For each of these traits, a reliable, validated self-report questionnaire exists. The identification of profiles might prove useful in a clinical setting, as they can be measured in an inexpensive, quick and relatively easy way. In BPD, very little is known about autistic traits and although several studies have investigated different aspects of emotional intelligence [4,14] and have looked at cognition, none have specifically investigated systemizing.

To measure the extent of autistic traits in any individual, we used the Autism Spectrum Quotient (AQ; [25]), an easy to administer instrument with good discriminative validity and screening properties [26]. The AQ has been used by many studies and norms are available for clinical and non-clinical groups from a systematic review of 78 studies, detailing 6,934 nonclinical participants as well as 1,963 matched clinical cases of ASC [27]. As a short and simple self-report questionnaire, it enabled us to achieve a high number of online responses.

In 2009, Baron-Cohen proposed the Empathising-Systemizing (E-S) theory of autism [28], based on the observation that people with ASC had below average empathy with average or above average systemizing. These can be measured using the Empathy Quotient (EQ; [11]) and the Systemizing Quotient (SQ; [24]. In the latter study of 47 adults with Asperger syndrome (AS) or high functioning autism (HFA) compared with 47 matched adults from the general population, adults with AS/HFA scored significantly higher on the SQ than matched controls, and significantly lower on the EQ than matched controls. This pattern of results was replicated in a much larger study [29].

In a comprehensive review [13] of the ‘borderline empathy paradox’, initially described by Krohn [30], both enhanced and impaired levels of empathy in BPD patients have been described. This paradox may reflect variations in the method used to measure empathy or state-based/situational factors (if the individual is stressed, their empathy is reduced). In addition, in BPD, reduced interpersonal trust may lead the individual to be hyper-vigilant about other’s facial expressions or tone of voice, leading to enhanced emotion recognition skills even if talking about other’s mental states (“why are you angry with me?”, or “are you fed up with me?”), which may be socially inappropriate. There is more consistent evidence supporting the borderline empathy paradox from more socially interactive experimental methods of assessment [31–33], as opposed to tests using more passive stimuli, such as the ‘Reading the Mind in the Eyes’ Test (RMET) [34–37]. This may reflect a greater sensitivity of interactive stimuli to test empathic enhancement in BPD patients, however further research in this area is required to better understand this.

Two studies have used self-report questionnaires to investigate empathy in BPD—both used the Interpersonal Reactivity Index (IRI) [38,39] and found a decrease in cognitive empathy. However, while Guttman & Laporte found increased affective empathy, Harari et al found there was no statistically significant difference compared to nonclinical controls. To our knowledge, the present study is the first to investigate systemizing in BPD. Also, as far as we are aware, no studies have investigated autistic traits, emotional intelligence or systemizing ability in patients who are comorbid with both ASC and BPD.

Recent research is focusing on the possible under-diagnosis or mis-diagnosis of ASC in females [40,41], on the assumption that females may experience greater societal pressure to conform and be part of a peer group and so are more motivated to learn how to hide their autism (so-called “camouflaging”) and thereby go “under the radar” of clinicians or school psychologists, by “pretending to be normal” [42]. This may lead to them either receiving their ASC diagnosis far later than males, and/or being diagnosed with other conditions (anorexia, depression, anxiety, or BPD) because clinicians are not looking for how ASC may present itself differently in females [43].

The current study can thus test if BPD and ASC share a common underlying cognitive phenotype (higher AQ, and SQ>EQ) irrespective of the clinical label they are given.

## Methods

### Participants

Participants were recruited from the Cambridge Autism Research Database (CARD), based at the Autism Research Centre, University of Cambridge. Participants with formal clinical diagnosis of ASC, according to DSM-IV or 5 or ICD-10 criteria, register online at www.autismresearchcentre.com and provide details of when and where they received their ASC diagnosis. Participants from the general population, without a formal diagnosis of ASC, register at a separate website (www.cambridgepsychology.com). All participants are asked to provide demographic details (age, education background, any clinical diagnoses), and complete a variety of self-report measures including the AQ, EQ and SQ-R.

*The Full Sample*. Data from a total of 2,744 online responders were analysed. 624 responders indicated that they had been diagnosed with an ASC, 23 with BPD, and 16 with both (ASC+BPD). 2,081 responders reported no diagnosis (NC). There were thus 4 groups in the study: BPD, ASC, BPD+ASC, and Controls.

*The Random Sample*. In order to circumvent the problem of unbalanced group sizes (and the assumption of homogeneity of variance being violated), we also tested our hypotheses in a smaller sample (N = 89). 25 ASC and 25 NC responders were randomly selected from our full sample.

Ethical approval for the research database was obtained from the Psychology Research Ethics Committee (PREC), University of Cambridge, UK. Consent was obtained online when participants registered to join the research database, where they have the opportunity to read the Terms and Conditions. This describes how the research data they provide (questionnaire and performance data) will be used in a variety of future research studies in an anonymised form, and that their personal information is only seen by named database managers who take legal responsibility for data protection.

### Instruments

#### The AQ

The Adult Autism-Spectrum Quotient (AQ) is a 50-item, self-report questionnaire for use with adults with normal intelligence to assess for the presence of traits associated with the autistic spectrum [25]. The questions assess 5 different areas, each consisting of 10 items on each: social skills, attention switching, attention to detail, communication, and imagination. Each AQ item is a brief statement followed by 4 possible ratings: “definitely agree,” “slightly agree,” “slightly disagree,” or “definitely disagree.” Each item is scored 1 point if the responder endorses the behaviour either mildly or strongly, resulting in a maximum possible score of 50. Higher AQ scores indicate a higher degree of autistic features, and a threshold of >26 is a good predictor of diagnosis [26].

#### The EQ

The Empathy Quotient (EQ) is a 60-item, self-report questionnaire for use with adults with normal intelligence for the quantitative measurement of empathy [11]. It consists of 40 empathy items and 20 filler/control items. On each of the empathy items a person may score 2, 1 or 0, which correlates with the responder reporting the behaviour strongly, mildly or not at all. Therefore the maximum score is 80 and the minimum is zero. 80% of people with ASC score <30.

#### The SQ-R

The Systemizing Quotient-Revised (SQ-R) is a 75-item, self-report questionnaire for use with adults with normal intelligence for the quantitative measurement of systemizing ability [44]. On 39 items, ‘strongly agree’ responses score two points and ‘slightly agree’ responses score one point, and on 36 items, ‘strongly disagree’ responses score two points and ‘slightly disagree’ responses score one point. The maximum score is 150 and the minimum is zero.

#### Brain types

The D score or ‘Brain Type’ is a measure of the standardized difference between an individual’s empathizing and systemizing scores. The raw SQ-R and EQ scores are standardized by subtracting the typical population mean (denoted by <…>) from the participant’s score and then dividing this by the maximum possible score (S = (SQ-R–<SQ-R>)/150 and E = (EQ–<EQ>)/80). The control group means are used as estimations of the typical population means in this standardization procedure: EQ (mean = 45.38, SD = 14.98) and SQ-R (mean = 58.55, SD = 22.34). The difference (D) between the standardized EQ and SQ-R scores is then calculated by: D = (S–E)/2. Using the D score, individuals can be classed into one of five cognitive profiles, or ‘brain types’. ‘Brain types’ based on D score are defined quantitatively, based on a previous study [45] that classed the lowest and highest 2.5^th^ percentiles of scores in a large, population-based, typically developing group as ‘Extreme Type E’ (E>>S) and ‘Extreme Type S’ (S>>E), respectively. Those scoring between the 2.5^th^ and 35^th^ percentiles are classed as ‘Type E’ (E>S), those between the 35^th^ and 65^th^ percentiles as ‘Type B’ (balanced, E≈S), and those between the 65^th^ and 97.5^th^ percentile as ‘Type S’ (S>E).

### Statistical analysis

We used a one-way analysis of variance (ANOVA) to compare means scores on dependent variables (AQ, EQ and SQ), between the four groups (Control, ASC, BPD, ASC+BPD), and post-hoc Games-Howell tests used to follow up significant main effects. We checked our findings with appropriate non-parametric tests (Kruskal-Wallis and Mann Whitney U tests) where our variables did not follow a normal distribution and their distribution could not be normalized by square root or logarithmic transformation. Cohen’s *d* was calculated as a measure of effect size for post-hoc comparisons, with > = 0.2 indicating a small, > = 0.5 a medium and > = 0.8 a large effect.

## Results

### Analysis of the full sample

The mean age of the sample was 39.43 years (SD = 12.3), and the 4 groups did not differ from each other on age (ANOVA F_3, 2740_ = -.395; p = 0.757, n.s.). The BPD group, and to a lesser extent the NC group, showed a female preponderance, whilst the ASC groups were well-balanced. Responders predominantly attended mainstream schools **(S1 Table)**.

#### The AQ scores

**Fig 1:**
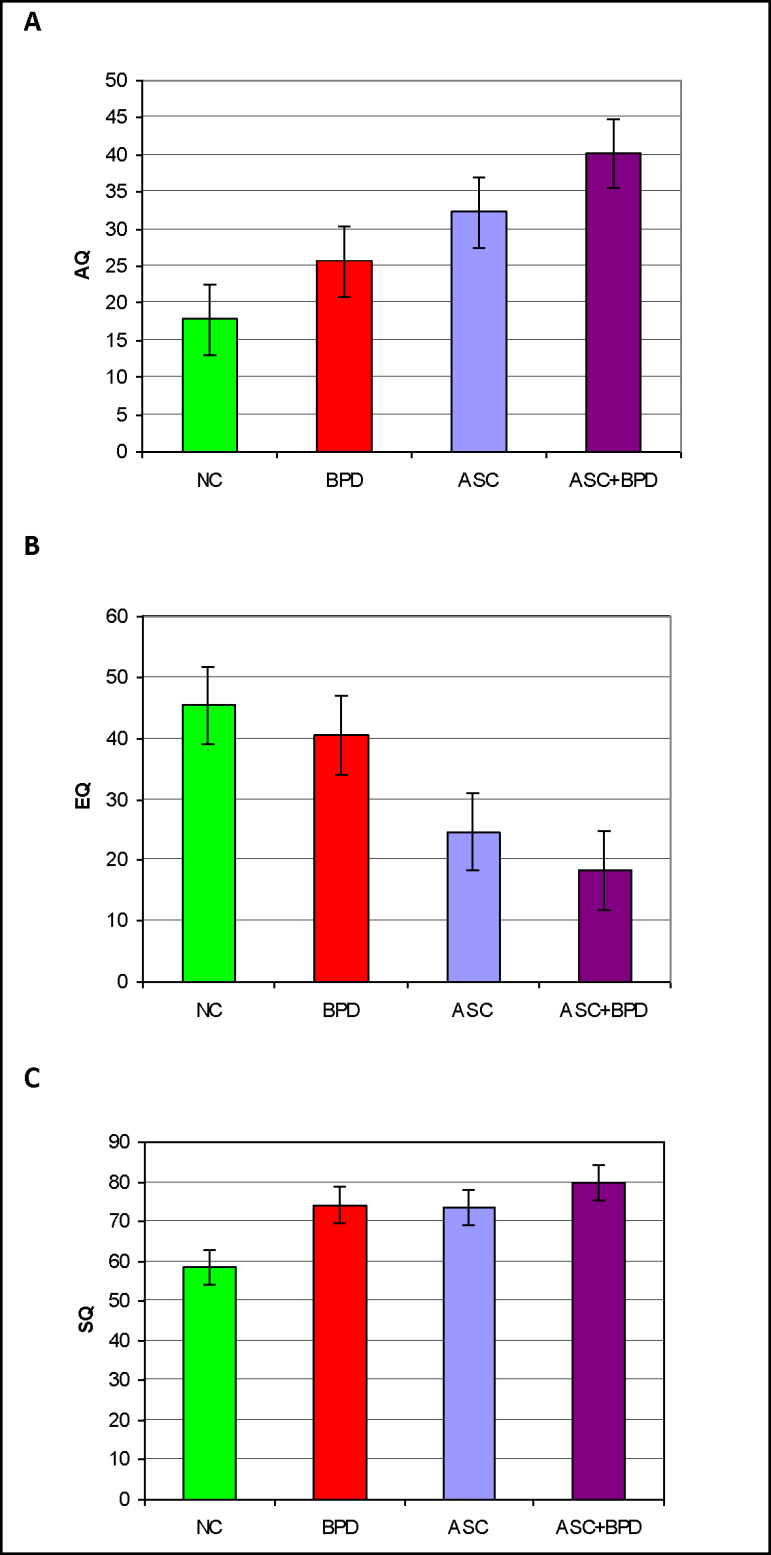
A Mean scores (and error bars) of the 4 diagnostic groups on the AQ in the full sample. B Mean scores (and error bars) of the 4 diagnostic groups on the EQ in the Full Sample. C Mean scores (and error bars) of the 4 diagnostic groups on the SQ-R in the Full Sample.

The AQ scores followed a normal distribution in the groups (**S2 Table**). Each group was significantly different from one another in terms of this measure (ANOVA F_3, 2727_ = 445.65; p < 0.001), resulting in the following pattern: NC < BPD < ASC < ASC+BPD (**Fig 1A**). Post hoc comparison with the Games-Howell test between the NC and BPD groups yielded a significant p value of 0.014 (Cohen’s d = 1.08), however, the difference between the BPD and the ASC group was only marginally significant (p = 0.047; Cohen’s d = 0.51). Patients with both conditions, the ASC+BPD group, scored highly significantly higher than those with ASC (p = 0.001; Cohen’s d = 0.71). The AQ scores of the two ASC patient groups very clearly separated them from the responders with no diagnosis (p < 0.001; Cohen’s d = 1.62 and 2.75, respectively for ASC and ASC+BPD).

#### The EQ scores

The EQ scores of the ASC+BPD group were positively skewed (skewness = 1.87, SE = 0.56; kurtosis = 3.07, SE = 1.09), and square root or logarithmic transformation only made the distributions less normal. A one-way ANOVA revealed highly significant between group differences (ANOVA F_3, 2424_ = 265.66; p < 0.001), with the BPD and NC not being different from each other but both scoring higher than the ASC and ASC+BPD groups (each p value < 0.001; Cohen’s d values: ASC vs NC = -1.37, ASC+BPD vs NC = -1.81, ASC vs BPD = -1.01, ASC+BPD vs BPD = -1.42), which were again not different from each other (Cohen’s d = -0.40; **Fig 1B**), resulting in the following pattern: NC = BPD > ASC = ASC+BPD. We checked our findings with the Kruskal-Wallis test, which was highly significant (p < 0.001). Mann Whitney U tests confirmed that the BPD and NC groups (p = .282) and the ASC and ASC+BPD groups (p = 0.05) respectively were not different from each other, but the NC and BPD groups scored significantly higher than the ASC group (p < 0.001 in both cases).

#### The SQ-R scores

The SQ-R scores were again relatively normally distributed and produced another pattern (ANOVA F3, 2326 = 62.51; p < 0.001), with both the BPD (Games-Howell: p = 0.016, Cohen’s d = 0.7) and the ASC (G-H: p < 0.001, Cohen’s d = 0.65) groups scoring significantly higher than the NC group: NC < BPD = ASC. The ASC+BPD group was not statistically different from any other group in our post hoc comparisons despite its mean being higher than that of any other group. However, Cohen’s d values indicated a large effect when looking at ASC+BPD compared to NC (d = 0.95), and a small effect when comparing the ASC+BPD group with the BPD (d = 0.22) or ASC (d = 0.24) groups, as suggested by a bar chart of the data (**Fig 1C**).

#### “Brain types”

**Fig 2:**
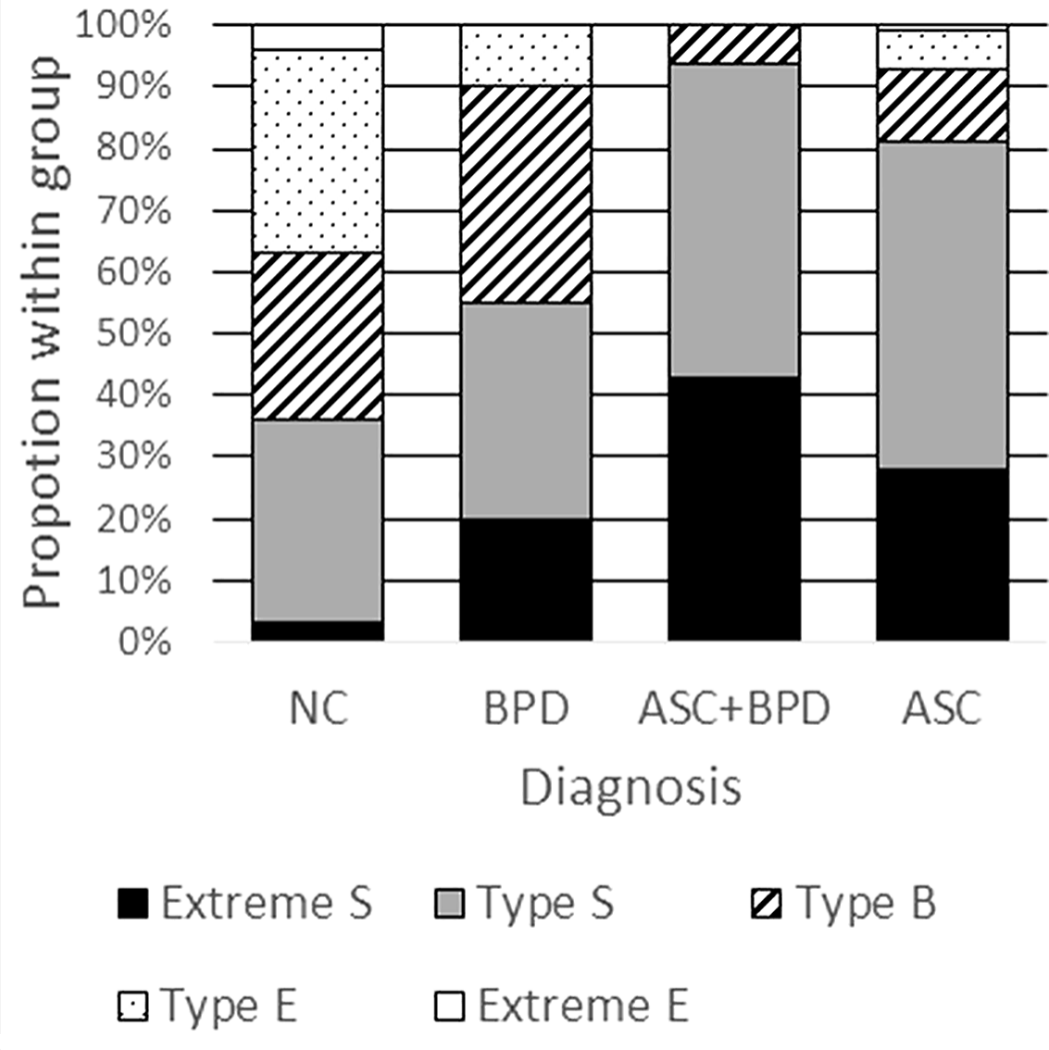
Proportion of brain types within the diagnostic groups.

An analysis of brain types revealed the expected pattern of distribution in the NC group (**Fig 2**). Approximately 80% of the ASC group had an “S” or “extreme S” type brain. Interestingly, over 50% of the BPD group also had “S” or “extreme S” type brains, largely due to a larger proportion of Extreme S brains relative to controls. By far the largest proportion (around 50%) of “extreme S” type brains was found in the ASC+BPD group.

### Analysis of the random sample

The groups were well-matched on age (ANOVA F_3, 85_ = 0.64; p = 0.592, n.s.). Generally, there was a female preponderance in the sample, with this being, again, particularly salient in the BPD and NC groups. Most responders attended mainstream school, but responders in the patient groups also reported having received education in special schools, their own homes, or via other arrangements (**S3 Table**).

#### The AQ scores

**Fig 3:**
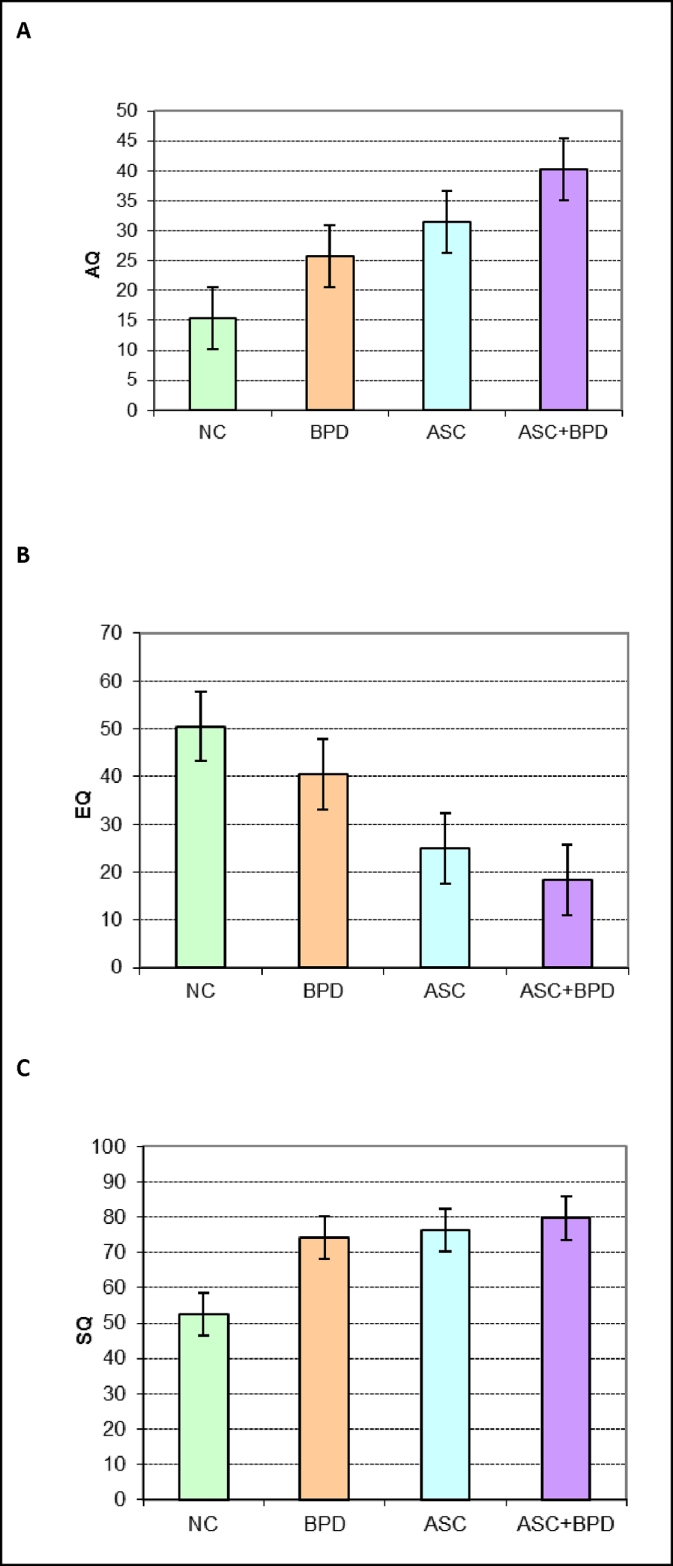
A Mean scores (and error bars) of the 4 diagnostic groups on the AQ in the random sample. B Mean scores (and error bars) of the 4 diagnostic groups on the EQ in the Random Sample. C Mean scores (and error bars) of the 4 diagnostic groups on the SQ-R in the Random Sample.

The AQ scores were normally distributed in the sample (**Fig 3A**, **S4 Table**). A one-way ANOVA demonstrated significant between-group differences (ANOVA F3, 85 = 18.52; p < 0.001) with the patient groups scoring significantly higher than NC group (BPD vs. NC: p = 0.011, Cohen’s d = 1.1) the ASC and ASC+BPD groups vs. NC: both p < 0.001, Cohen’s d = 1.51 and 2.68, respectively). In this random sample, the mean score of the BPD group was not statistically different from that of the ASC group (Cohen’s d = 0.41). The ASC+BPD group scored higher than the NC and BPD groups, but its slightly higher mean score was not statistically different from that of the ASC group (p = 0.084, Cohen’s d = 0.63). These results were a partial replication of the pattern seen in the Full Sample: NC < BPD, BPD < ASC+BPD, (BPD = ASC).

#### The EQ scores

The EQ scores were non-normally distributed and were analysed following square root transformation. A one-way ANOVA revealed significant between-group differences (F_3, 74_ = 15.75; p < 0.001), with the BPD and NC not being different from each other (NB. Cohen’s d = -0.61) but both scoring higher than the ASC and ASC+BPD groups (each p value < 0.001, Cohen’s d: BPD vs ASC = -0.84, BPD vs ASC+BPD = -1.42, ASC vs NC = -1.5, ASC+BPD vs NC = -1.95), which were again not different from each other (Cohen’s d = -0.37, **Fig 3B**). This was the same pattern as observed in the Full Sample: NC = BPD >> ASC = ASC+BPD.

#### The SQ-R scores

The SQ-R scores were non-normally distributed and therefore analysed after square root transformation. A one-way ANOVA indicated significant between-group differences (F3, 74 = 4.48; p = 0.006) with the patient groups all scoring significantly higher than the NC group (BPD vs. NC: p = 0.032, Cohen’s d = 0.76; ASC vs. NC: p = 0.025, Cohen’s d = 0.83; ASC+BPD vs NC: p = 0.022, Cohen’s d = 0.95). This pattern (NC < BPD = ASC = ASC+BPD) was similar to that found in the Full Sample (**Fig 3C**).

We summarized our findings in **Table 1**.

**Table 1:** Autistic traits, empathizing ability and systemizing ability in ASC, BPD, and comorbid patients relative to controls.

	BPD	ASC	ASC+BPD
**AQ**	↑	↑	↑
**EQ**	↔	↓	↓
**SQ**	↑	↑	(↑)

The symbol in brackets indicates findings from the random sample when different from the full sample.

## Discussion

To our knowledge, this is the first study to investigate autistic traits, empathizing and systemizing abilities in ASC, BPD, comorbid ASC+BPD, and controls. The mean AQ scores found in our ASC and control groups were comparable to those reported by a recent systematic review for nonclinical populations (16.94, 95% CI 11.6, 20.0) and for ASC (35.19, 95% CI 27.6, 41.1) [27]. Our finding of the comorbid ASC+BPD group scoring higher than the ASC group was consistent with a previous study reporting higher AQ scores in people with Asperger Syndrome who also met criteria for a personality disorder [17]. We are only aware of one previous study that reported results with the AQ in people with BPD [46]. Out of 38 women with BPD, almost half of them scored above the cut-off of the AQ. The difference on the AQ between the BPD and the ASC group was not statistically significant in our random sample, suggesting that people with BPD may have as high levels of autistic traits as people with ASC. This finding would need to be replicated in a larger sample but is in line with the idea that some females with BPD have undiagnosed ASC, due to ASC not being easily detected in females.

As expected, people with an ASC scored lower than controls on empathizing. The finding of no difference between people with BPD and controls on empathizing ability is consistent with one [39] but not another previous study [38] that used a self-report questionnaire to examine empathy.

As expected, participants reporting an ASC diagnosis on average reported higher levels of systemizing than those without such a diagnosis. A trade-off between empathizing and systemizing has been proposed, with ASC patients showing below average empathizing but intact or superior systemizing [44]. Our “brain type” analysis suggested a shift in those with BPD (either alone or comorbid with ASC) toward having a more systemizing type brain. As far as we are aware, no previous study has investigated systemizing in BPD. Our findings suggest that people with BPD also report elevated systemizing relative to controls, without a statistically significant difference between them and the ASC groups. It is possible that increased systemizing might be a compensatory mechanism for their emotional instability but, alternatively, elevated systemizing may be part of the phenomenology of BPD, just as it is in ASC. Our findings highlight the need for careful examination for autistic traits in patients referred for an assessment for BPD, especially in those without a history of significant childhood abuse or neglect. Some of these patients may have been misdiagnosed (their ASC was overlooked), and a proportion of them may have both conditions.

This study has two key limitations: the small size of the BPD and ASC+BPD groups, and that diagnosis was based on self-report. Self-report is common in large, online samples and is unlikely to be responsible for the differences found, as the group means in the ASC and NC groups were very similar to those derived from samples with a clinically verified diagnosis. The current findings need to be replicated, ideally using experimental paradigms that are more able to control for current mood state and less susceptible to bias in memory [47] and lack of insight. Finally, future research may investigate patterns with these instruments in other personality disorders, such as schizoid, schizotypal, and antisocial personality disorder.

## Supporting information

S1 Table Demographic variables in the 4 diagnostic groups of the full sample. * mainstream:special:other:home.
(DOCX)

S2 Table AQ, EQ, and SQ-R mean scores in the NC, BPD, ASC, and ASC+BPD groups in the full sample. Values are means (and standard deviations).
(DOCX)

S3 Table Demographic variables in the four diagnostic groups of the random sample. * mainstream:special:other:home.
(DOCX)

S4 Table AQ, EQ, and SQ-R mean scores in the NC, BPD, ASC, and ASC+BPD groups in the random sample. Values are means (and standard deviations).
(DOCX)
