# Correction: The overlap between autistic spectrum conditions and borderline personality disorder

**DOI:** 10.1371/journal.pone.0190727

**Published:** 2018-01-02

**Authors:** Robert B. Dudas, Chris Lovejoy, Sarah Cassidy, Carrie Allison, Paula Smith, Simon Baron-Cohen

There is an error in the first sentence of the Results section of the Abstract. The correct sentence is: On the AQ, the comorbid group scored higher than the ASC group, who in turn scored higher than the BPD group, who scored higher than controls.
