# Supplementary material for: The overlap between autistic spectrum conditions and borderline personality disorder
Source: PLoS One. 2017 Sep 8;12(9):e0184447. doi: 10.1371/journal.pone.0184447 (PMC5590952; doi:10.1371/journal.pone.0184447)
Supplement: S1 Table — * mainstream:special:other:home. (DOCX) [file pone.0184447.s001.docx]

**Supplemental Table 1.** Demographic variables in the 4 diagnostic groups of the Full Sample

|  | **NC** | **BPD** | **ASC** | **ASC+BPD** | **ANOVA F** |
| --- | --- | --- | --- | --- | --- |
| **Age**  mean (SD) | 39.48  (12.3) | 38.83  (9.26) | 39.36  (13.3) | 36.19  (11.62) | 0.395 |
| **Sex** (Male:Female) | 696:1386 | 3:20 | 313:311 | 7:9 | - |
| **School*** | 1945:36:90:9 | 18:1:2:2 | 511:24:34:5 | 13:0:1:2 | - |

* mainstream:special:other:home
