# Supplementary material for: The overlap between autistic spectrum conditions and borderline personality disorder
Source: PLoS One. 2017 Sep 8;12(9):e0184447. doi: 10.1371/journal.pone.0184447 (PMC5590952; doi:10.1371/journal.pone.0184447)
Supplement: S2 Table — Values are means (and standard deviations). (DOCX) [file pone.0184447.s002.docx]

**Supplemental Table 2.** AQ, EQ, and SQ-R mean scores in the NC, BPD, ASC, and ASC+BPD groups in the Full Sample

|  | **NC** | **BPD** | **ASC** | **ASC+BPD** | **ANOVA F** |
| --- | --- | --- | --- | --- | --- |
| **AQ** | 17.79  (8.15) | 25.65  (11.26) | 32.32  (11.21) | 40.19  (6.07) | 445.65 |
| **EQ** | 45.38  (14.98) | 40.45  (17.35) | 24.6  (15.73) | 18.31  (13.88) | 265.66 |
| **SQ** | 58.55  (22.34) | 74.19  (21.49) | 73.68  (25.57) | 79.73  (31.39) | 62.51 |

Values are means (and standard deviations).
