# Supplementary material for: The overlap between autistic spectrum conditions and borderline personality disorder
Source: PLoS One. 2017 Sep 8;12(9):e0184447. doi: 10.1371/journal.pone.0184447 (PMC5590952; doi:10.1371/journal.pone.0184447)
Supplement: S3 Table — * mainstream:special:other:home. (DOCX) [file pone.0184447.s003.docx]

**Supplemental Table 3.** Demographic variables in the four diagnostic groups of the Random Sample

|  | **NC** | **BPD** | **ASC** | **ASC+BPD** | **ANOVA F** |
| --- | --- | --- | --- | --- | --- |
| **Age**  mean (SD) | 36.04  (10.27) | 38.83  (9.26) | 39.84  (13.73) | 36.19  (11.62) | 0.64 |
| **Sex (Male:Female)** | 7:18 | 3:20 | 12:13 | 7:9 | - |
| **School type*** | 25:0:0:0 | 18:1:2:2 | 16:4:4:0 | 13:0:1:2 | - |

* mainstream:special:other:home
